# Expression of concern: Enhancement of anti-leukemic potential of 2-hydroxyphenyl-azo-2′-naphthol (HPAN) on MOLT-4 cells through conjugation with Cu(ii)

**DOI:** 10.1039/d4ra90056a

**Published:** 2024-05-15

**Authors:** Tathagata Deb, Priya Kalyan Gopal, Durba Ganguly, Piyal Das, Mausumi Paul, Manju Bikash Saha, Santanu Paul, Saurabh Das

**Affiliations:** a Department of Chemistry (Inorganic Section), Jadavpur University Kolkata 700 032 India dasrsv@yahoo.in +91 33 24146223 +91 8902087756; b Laboratory of Experimental Immunology, Department of Microbiology and Botany, Gurudas College Kolkata 700 054 India spaul_1971@yahoo.com +91 33 23546623 +91 9874192648; c Department of Chemistry, Indian Institute of Chemical Biology Kolkata 700 032 India

## Abstract

Expression of concern for ‘Enhancement of anti-leukemic potential of 2-hydroxyphenyl-azo-2′-naphthol (HPAN) on MOLT-4 cells through conjugation with Cu(ii)’ by Tathagata Deb *et al.*, *RSC Adv.*, 2014, **4**, 18419–18430, https://doi.org/10.1039/C3RA44765K.

*RSC Advances* is publishing this expression of concern to alert readers that we are presently unable to confirm the reliability of the data presented in the microscopic images in Fig 4 of this article.

The Royal Society of Chemistry has asked the affiliated institution (Gurudas College) to investigate this matter and confirm the integrity and reliability of the data in Fig. 4 of the paper. An expression of concern will continue to be associated with this manuscript until we receive information from the institution on this matter.

Signed: Laura Fisher, Executive Editor, *RSC Advances*

Date: 9th May 2024

## Supplementary Material

